# Allicin treats myocardial infarction in I/R through the promotion of the SHP2 axis to inhibit p-PERK-mediated oxidative stress

**DOI:** 10.18632/aging.205640

**Published:** 2024-03-07

**Authors:** Tong Gao, Mengru Liu, Dongliang Fu, Yajun Xue, Jiangquan Liao, Peng Yang, Xianlun Li

**Affiliations:** 1 Department of Cardiology, Beijing Tsinghua Changgung Hospital, Medical Center, Tsinghua University, Beijing 102218, China; 2 Department of Integrative Medicine Cardiology, China-Japan Friendship Hospital, Beijing 100029, China

**Keywords:** allicin, SHP2, I/R, oxidative stress, NLRP3

## Abstract

Objective: The study attempted to explore how allicin reduces oxidative stress levels by promoting SHP2 expression to inhibit p-PERK in I/R mice.Methods: The GEO database and RNA sequencing were used to predict downstream gene. TTC staining was used to visualize the myocardial infarction area. Masson staining was used to assess the level of fibrosis. IF was used to examine the expression of SHP2, CTGF, ROS. RT-PCR analysis was used to quantify the expression of SHP2 mRNA. Western blot was used to detect the protein expression levels of SHP2, p-PERK, MFN1, NLRP3, NOX2, and NOX3.

Results: GEO and transcriptomic data revealed low expression of SHP2 in the heart tissues I/R mice. In the I/R mouse model, TTC staining result showed that allicin can reduce the area of myocardial infarction; Masson staining results indicated that allicin can reduce fibrosis; Macrophage transcriptome sequencing found SHP2 is a target gene of allicin; Immunofluorescence showed allicin can increase SHP2; qPCR results showed allicin can raise SHP2 mRNA level; Immunofluorescence indicated that allicin can inhibit ROS in myocardial infarction tissue, but the specific SHP2-KD eliminates changes in ROS. Western blot analysis demonstrated allicin can increase SHP2 protein and reduce the expression of p-PERK, MFN1, NLRP3, NOX2, and NOX3; SHP2-KD eliminates the expression differences in p-PERK, MFN1, NLRP3, NOX2, and NOX3.

Conclusions: Allicin can modulate p-PERK activation by enhancing the expression of SHP2, thereby inhibiting myocardial ischemia-reperfusion-induced oxidative stress in mice.

## INTRODUCTION

Acute myocardial ischemia is mostly caused by the rapid worsening of coronary heart disease and the acute persistent insufficiency of coronary blood supply, characterized by rapid and critical onset, which is a common emergency critical disease [1]. The incidence of ischemic cardiovascular disease is increasing and shows a younger trend, seriously threatening human life and health [2]. Coronary artery recanalization by various technical means it is the preferred regimen in the clinical treatment of patients with acute myocardial infarction but decreased cardiac function and malignant arrhythmia, even sudden cardiac death may be further caused after reperfusion. Therefore, it has been a problem demanding prompt solutions to prevent and treat myocardial ischemia/reperfusion injury (MIRI) in clinical treatment of ischemic cardiovascular disease [1].

First, we understand the prevention mechanism and pathophysiological basis of MIRI [3]. Mitochondria-associated endoplasmic reticulum membrane (MAM) is a dynamic structure between mitochondria and endoplasmic reticulum, which can regulate the functions of mitochondria and endoplasmic reticulum [4]. There is growing evidence that MAM is an important player in energy generation, cell contraction and movement, and intracellular/extracellular signal transmission, and it can be implicated in such biological processes as accumulation of mitochondrial reactive oxygen species (ROS), release of downstream inflammatory factors, regulation of autophagy, endoplasmic reticulum stress and programmed cell death. As a storage place for intracellular Ca^2+^, the endoplasmic reticulum can respond to intracellular electrical or chemical stimuli through releasing or recycling Ca^2+^. Ca^2+^-mediated signal transduction is conducive to the rapid response of cells to external stimuli, thereby maintaining homeostasis [5]. Mitochondria are organelles for intracellular energy metabolism, and Ca^2+^ is an agonist of a variety of enzymes in the mitochondrial oxidative respiratory chain, which can determine the generation rate of adenosine triphosphate (ATP) [6]. I/R can lead to depolarization of cell membrane potential and opening of voltage-gated Ca^2+^ channels, so that a large amount of Ca^2+^ enters the cell to break Ca^2+^ homeostasis and cause mitochondrial Ca^2+^ overload, Then the generation of ATP is decreased, while the production of ROS is increased, and the mitochondrial permeability transition pore (MPTP) channel opens, thus resulting in mitochondrial swelling and apoptosis [7, 8]. Therefore, the oxidative stress induced by Ca^2+^ homeostasis disorders in MAM plays a key role in the pathogenesis of I/R. Oxidative stress (OS) leads to an imbalance between oxidation and oxidation, resulting in inflammatory neutrophil infiltration, enhanced protease secretion, and massive production of oxidative intermediates. Considering that SHP2 is a key inhibitor for NLRP3 activation [9] and is the modulator for SHP2 [10], thus the relationship between SHP2 and NLRP3 as well as ROS was used in this study.

Allicin, also known as s-allyl-2-ene-1-thiosulfite, is one of the major biologically active compounds in allicin [11], with a wide range of pharmacological properties, including anti-inflammation, immunoregulation, anti-oxidation, renal protection, nerve protection, cardiac protection and anti-tumor activity [12]. It has been confirmed that allicin and its derivatives, such as organosulfur compounds, exert biological effects through interaction with many signaling pathways and alteration in gene expression [13]. There have been broad reports on the beneficial effect of allicin in the treatment of cardiovascular, neuroinflammatory and neurodegenerative diseases [14, 15], but its specific mechanism remains unknown. Therefore, the MIRI mice model was established using macrophage-specific conditional SHP2-knockout (cSHP2-KO) mice, and the regulatory effect of allicin on SHP2 and its specific mechanism in mouse I/R was explored through a series of *in vivo* and *in vitro* experiments.

## MATERIALS AND METHODS

### Bioinformatics analysis

We searched for MIR-related datasets from the Gene Expression Comprehensive Database (GEO) database (https://www.ncbi.nlm.nih.gov/gds/). We looked for the datasets GSE171821 and GSE108940 for MIR-related gene expression and downloaded them. Quantile normalization of RNA-seq data was done using the limma software package of R software, and the differentially expressed genes (DEGs) were analyzed (|logFC|<1, p<0.05). Moreover, visually grouped volcano maps of DEGs in GSE171821 and GSE108940 were constructed using the ggplot2 package of R software. The clustering heat map of DEGs was plotted using the dynamic plot package of R software.

### Functional enrichment analysis

The RobustRankAggreg software package of R software was used to cross-process GSE171821 and GSE108940 to obtain generic DEGs. These DEGs were analyzed for enrichment by Gene Ontology (GO) and the Kyoto Encyclopedia of Genes and Genomes (KEGG). The online database tool DAVID (https://david.ncifcrf.gov) was used to analyze DEGs at the level of biological processes, cellular components, and molecular functions, integrate GO terminology, and create a bioprocess network of DEGs. The GOplot and ggplot2 software packages of R software were used to plot the GO and KEGG pathway enrichment analysis of DEGs.

### Protein-protein interaction (PPI) network analysis and target gene screening of DEGs

DEGs were entered into an online string of tools to screen for interacting proteins with an overall score of >0.9. Then the PPI results obtained were imported into Cytoscape software, and the target genes with a score <10 were obtained using the plugin cytoHubba and degree algorithm.

### Gene set enrichment analysis (GSEA)

GSEA analysis was performed on all genes using R Software’s Cluster Analyzer software package, and GSEA pathway enrichment analysis maps were plotted.

### Animal modeling and grouping

The cSHP2-KO and wild-type (WT) mice will be presented to Dr. Lu Yonggang. The MIRI model was established using the method in the literature [13]. Preparing the surgical equipment required for the experiment: 10% hydrate solution, 1 ml syringes, povidone-iodine, cotton swabs, electric hair clippers, surgical board, 4 rubber bands, scissors, 2 large ophthalmic curved forceps, 1 needle holder, 1 hemostat, 6-0 suture thread with needle, 3-0 suture thread, suture needle, electronic scale, ventilator. Firstly, anesthetize the mice, with commonly used anesthetics such as pentobarbital sodium solution, typically at a dosage of 50 mg/kg body weight. Make a 4 cm long vertical skin incision on the left side of the sternum, bluntly separate the subcutaneous muscles, and expose the pleura between the third and fourth ribs. After entering the thoracic cavity by tearing the pleura with forceps, use the hemostat to pry the ribs apart, exposing the heart. Gently tear the pericardium with the forceps and cautiously clamp the heart out with the hemostat (avoid touching the lungs during operation). Ligate with a 6-0 suture thread (the ligation site is parallel with the tip of the left atrial appendage), softly place the heart back into the thoracic cavity. Check for any bleeding inside the heart. After confirming there is no bleeding, suture layer by layer with 3-0 suture thread. Disinfect with povidone-iodine after suturing, then remove the ventilator and place the rat back in the cage. Use a chest opener to expose the heart, separating the surrounding pericardium. Ligate the left anterior descending coronary artery 2-3 mm below the left atrial appendage to induce localized myocardial ischemia. Place a small pad between the ligatures to prevent myocardial laceration. Wait until the color of the left ventricular anterior wall tissue changes from red to pale white, indicating successful ischemia. About 30 minutes after the onset of ischemia, reopen the chest cavity, cut the ligature to restore blood flow and achieve reperfusion. After removing the ligature, close the thoracic cavity and suture the skin. Harvest cardiac tissue samples for further analysis 24 hours after reperfusion [16].

### 2,3,5-triphenyl-2H-tetrazolium chloride (TTC) staining

We used 2,3,5-triphenyl-2H-tetrazolium chloride (TTC) staining to detect myocardial infarction. TTC solution was prepared immediately before use in a phosphate buffer solution (PBS, pH = 7.4) in darkness at 37° C. After washing with PBS solution, cut the mouse heart into 5 short-axis sections from apex to base. Mice hearts are stained with TTC (1%) solution at 37° C for 15 min [17]. The images of each group of stained sections were collected, and the infarct area and total cross-section area were measured by ImageJ software.

### Masson staining of myocardial tissues

After deparaffinization, the paraffin sections were immersed in mordant, and dried in a constant temperature incubator at 60° C for 1 h [15]. After washing, the sections were instilled with celestine blue and Mayer’s hematoxylin, differentiated with acidic ethanol, instilled with Ponceau S, washed with water, and added with phosphomolybdic acid hydrate. After the liquid was poured away, the sections were added with aniline blue, washed with weak acid, dehydrated with 95% ethanol and absolute ethanol, naturally dried, mounted with neutral balsam, and covered with a cover glass. The changes in collagen fibers in myocardial tissues were observed and photographed under a microscope. Finally, the myocardial fibrosis region was measured using Image Pro-Plus-6 software (Media Cybernetics).

### RT-PCR

Total RNA in cardiac tissue was extracted with TRIzol and synthesized into cDNA using a reverse transcriptase kit. Then qPCR was performed in a real-time system (Bio-Rad, USA) using TaqMan Reverse Transcription Kit (N8080234, Thermo Fisher Scientific, USA). The reaction conditions are as follows: pre-denaturation (95° C, 3 min), (95° C, 5 s), (56° C, 10 s), (72° C, 25 s) × 40 cycles. Primers are as follows: SHP2 (F: 5’-TCTATGGTGGGGAGAAGTTTGC-3’, R: 5’-ACAGTTCAGCGGGTACTTGAG-3’), GAPDH (F: 5’-TCAACGGCACAGTCAAGG-3’, R: 5’-TCAACGGCACAGTCAAGG-3’). The results were analyzed using the 2^-ΔΔ Ct^ method.

### Immunofluorescence

Immunofluorescence staining was conducted every 6 sections of heart tissues, and p-SHP2 and macrophage CD68 were positioned and semi-quantitatively detected. The tissue sections were incubated with polyclonal antibodies at 37° C for 60 min, then at 4° C overnight with TRITC-conjugated anti-rabbit immunoglobulin for 60 min at room temperature [17]. Finally, the expression changes of SHP2 and cardiac troponin T and CTGF were detected under a fluorescence microscope.

### Reactive oxygen species assay

### Loading the probe

For cells stimulated for a shorter duration (usually less than 2 hours), load the probe first, then stimulate the cells with reactive oxygen species positive control or the drug of interest. For cells stimulated for a longer duration (typically more than 6 hours), stimulate the cells with a reactive oxygen species positive control or the drug of interest first, then load the probe. *In situ* probe loading: This method is only applicable to adherent cultured cells. Dilute DCFH-DA with serum-free culture medium at a ratio of 1:1000 to achieve a final concentration of 10μM. Remove the cell culture medium and add an appropriate volume of diluted DCFH-DA to sufficiently cover the cells, typically adding no less than 1 mL of diluted DCFH-DA to one well of a six-well plate. Incubate in a 37° C cell culture incubator for 20 minutes. Wash the cells three times with serum-free culture medium to remove any DCFH-DA that did not enter the cells. Generally, reactive oxygen species positive control can significantly increase the level of reactive oxygen species in the cells after 20-30 minutes of stimulation. Post-collection probe loading: Dilute DCFH-DA with serum-free culture medium at a ratio of 1:1000 to a final concentration of 10 μM. After cell collection, suspend the cells in the diluted DCFH-DA with a cell density of one to two million/mL and incubate them in a 37° C cell culture incubator for 20 minutes. Mix by inverting every 3-5 minutes to ensure full contact between the probe and the cells. Wash the cells three times with serum-free culture medium to fully remove any DCFH-DA that did not enter the cells. Directly stimulate cells with reactive oxygen species positive control or the drug of interest, or divide the cells into several portions before stimulation. Generally, reactive oxygen species positive control can significantly elevate reactive oxygen levels in the cells after 20-30 minutes of stimulation.

### Mitochondrial probe detection

Preparation of JC-1 staining working solution: For each well of a six-well plate, 1mL of JC-1 staining working solution is needed, and the amount for other culture vessels can be deduced accordingly; for cell suspensions, 0.5mL of JC-1 staining working solution is required for every 50–100 thousand cells. Take an appropriate amount of JC-1 (200×) and dilute it with ultrapure water at the ratio of 50μL JC-1 (200×) to 8mL water. Vigorously shake to completely dissolve and mix JC-1. Then add 2mL of JC-1 staining buffer (5×) and mix well to obtain the JC-1 staining working solution. Setting up the positive control: Add CCCP (10mM), which is provided in the kit, to the cell culture medium at a recommended dilution ratio of 1:1000 to a final concentration of 10μM, and treat the cells for 20 minutes. Afterward, load JC-1 following the method described below to detect mitochondrial membrane potential. For most cells, the mitochondrial membrane potential will be completely lost after treatment with 10μM CCCP for 20 minutes, and JC-1 staining should show green fluorescence; whereas normal cells should show red fluorescence after JC-1 staining. For specific cells, the working concentration and time for CCCP may vary.

1. For one well of a six-well plate, remove the culture medium. If necessary for the specific experiment, wash the cells once with PBS or another appropriate solution, and add 1mL of cell culture medium. The culture medium can contain serum and phenol red. 2. Add 1mL of the JC-1 staining working solution and mix thoroughly. Incubate at 37° C in a cell culture incubator for 20 minutes. 3. During the incubation period, prepare an adequate amount of JC-1 staining buffer (1×) at a ratio of 1mL JC-1 staining buffer (5×) to 4mL distilled water and place it on ice. 4. After the 37° C incubation, aspirate the supernatant and wash twice with JC-1 staining buffer (1×). 5. Add 2mL of cell culture medium, which can contain serum and phenol red. 6. Observe with a fluorescence microscope. Flow cytometric sorting of cardiac resident macrophages was done.

Sodium pentobarbital intraperitoneal anesthesia, soaking in 75% ethanol, placed on an ultra-clean workbench, fixed onto the dissection board; the neck skin is incised, the thorax is opened, and 1 ml of PBS is injected into the heart using a sterile syringe, repeating 3 times to flush the pulmonary circulation in the blood, carefully removing the thoracic glands and thoracic lesions, and excising the entire lung at 4° C containing 5% fetal bovine serum (FBS) RPMI-1640; with ophthalmic scissors, cut and transfer to a 15 ml centrifuge tube, spin at 1500 rpm for 5 minutes, discard the supernatant, add 10 ml of digestion buffer (0.5 mg/ml collagenase IV, 0.02 mg/ml DNase I), incubate in 37° C water bath for 30 minutes, shaking every 5 minutes; triturate tissue, add another 5 ml of digestion buffer, digest for an additional 15 minutes; pass the cell mixture consecutively through a syringe to break up tissue clumps, use a TXM cell strainer to obtain a single cell suspension for cells over 70. Centrifuge at 1500 rpm for 5 minutes in PBS containing no Ca^2+^, Mg^2+^, with 10 mmol/L ethylenediaminetetraacetic acid (EDTA), resuspend at room temperature with agitation for 5 minutes; centrifuge again at 1500 rpm for 5 minutes; discard the supernatant, add 5 ml of red blood cell lysis buffer, ice lysing for 10 minutes; add 5 ml of RPMI-1640 containing 10% FBS to terminate lysis at 4° C. Centrifuge at 1200 rpm for 10 minutes to collect the cells. CD11b and CD11c antibodies are used for labeling cardiac single cell suspensions. After cell count, resuspend in flow buffer (RPMI-1640 + 1% FBS) typically 1×10^4^ cells in 300 μl of buffer; add 3 μl of FC blocking antibody, incubate at room temperature for 15 minutes. Divide the cell suspension into two tubes. Tube 1 serves as the negative control; to tube 2 add anti-CD11b-APC (or CD11b-PE-CY7) and 3 μl anti-CD11c-PE antibodies to constitute the test group, in the dark, label at 4° C for 20 minutes; then wash twice with flow buffer, then resuspend in 2.5 ml of flow buffer and transfer to a flow tube for flow cytometric sorting. This experiment uses the BD factorial flow cytometer, with parameters adjusted and sorting conditions set for sorting. RT-PCR is used to identify the macrophage marker CD68.

### Macrophage transcriptome sequencing

### Sample preparation

Six samples each from the control group, model group, and treatment group containing the same number of cells are extracted and quantified for RNA using the PicoPure RNA Isolation Kit (Arcturus, KIT0204, Life Technologies, USA), stored at -80° C. The quality and purity of RNA are assessed using the Agilent 2100 Bioanalyzer.

### cDNA library construction

Total RNA is processed using either mRNA enrichment or rRNA removal methods. For mRNA enrichment: Polyadenylated mRNAs are enriched using Oligo(dT) magnetic beads. For rRNA removal: rRNA is hybridized with DNA probes, followed by selective cleavage of DNA/RNA hybrids by RNase H, and cleavage of the DNA probes with DNase I to purify the desired RNA. The resulting RNA is fragmented using fragmentation buffer and reverse transcribed using random N6 primers, then synthesized into cDNA double-strand, forming double-stranded DNA. The ends of the synthesized dsDNA are repaired and phosphorylated at the 5’ end to create a blunt end, “A-tailing” at the 3’ end, and then ligated with a bubble adaptor with a protruding “T” at the 3’ end. PCR amplification is performed using specific primers. The PCR product is denatured to single-strand, and then single-stranded DNA is circularized with bridge primers to obtain a single-stranded circular DNA library. Computer sorting was done.

### Sequencing data filtering

Raw sequencing data are filtered to remove low-quality reads (bases with a quality score less than 15 account for more than 20% of the reads), adapter contaminations, and reads with a high percentage of unknown bases N. This produces clean reads, which are then quality-assessed, where the evaluation criteria primarily include: Q20 (proportion of bases with a quality value greater than 20 in filtered reads), Q30 (proportion of bases with a quality value greater than 30 in filtered reads), and GC content. After filtering, sequencing data reliability and the accuracy of data analysis results are ensured.

### Reference genome alignment

After obtaining clean reads, we align them to the reference genome sequence using HISAT. The average mapping rate per sample is obtained, and consistent mapping rates across samples indicate the comparability of the data. FDR must not exceed 0.05. With the obtained FDR values for differential testing, we calculate the differential expression multiples between samples based on the gene expression levels (FPKM values). The smaller the FDR value and the higher the multiple, the more significant the expression difference. In our analysis, differentially expressed genes are defined by default as those with an FDR ≤ 0.001 and a fold change of 2 or more.

### Distribution of gene expression in samples

To visually present the number of genes in different FPKM ranges for each sample, the numbers of expressed genes for three different FPKM categories (FPKM≤1, FPKM1~10, FPKM≥10) are counted based on the gene expression information.

### Differential gene expression screening

Based on the gene expression levels of each sample, differentially expressed genes (DEGs) between samples (sample groups) are detected. We employ the PossionDis method for differential detection and use RSEM software for quantitative analysis of alignment results, estimating gene expression levels with the FPKM method. After normalizing the read count with TMM, differential expression studies and analyses are conducted using the DESeq algorithm with q<0.005 and |log2 Fold Change|>1 as the screening criteria for differential gene function. According to demands, we detect differential genes with the PossionDis algorithm based on the principle of Poisson distribution. Then, p-values from differential testing are corrected with multiple hypothesis testing adjustments, controlling the FDR (False Discovery Rate) to determine the threshold. If R differentially expressed genes are selected, S of which are truly differentially expressed and V are false positives, we want the average proportion of errors Q = V/R to not exceed a tolerable value (for example, 5%).

### Differential gene GO functional analysis

Based on the results of differential gene detection, Gene Ontology (GO) functions are classified and enrichment analyzed. GO is divided into molecular function, cellular component, and biological process, and each category is further classified and enriched. Significant enrichment of genes is evaluated using an FDR<0.05 and P-value<0.05. According to the GO annotation results and classifications, differentially expressed genes are functionally categorized, and enrichment analysis is performed using the hyper function in software. Then, the p-values are corrected for FDR, and functions with an FDR ≤ 0.01 are considered significantly enriched.

### Differential gene pathway functional analysis

Pathway analysis returns a p-value for each pathway with differentially expressed genes, where a small p-value suggests enrichment of differential genes in that pathway, and the rate of false judgment is calculated. Pathway analysis is indicative of the experimental results; by analyzing the differential gene pathways, significant and targeted pathways related to the main expression trends are identified. Differences in gene expression between different samples might be related to changes in cellular pathways, with up and down-regulated differential genes mapped onto each pathway map to locate key genes in critical pathways. To study gene function, KEGG pathway enrichment analysis is performed on differential genes to annotate the biochemical metabolism pathways and signal transduction pathways involved. The differential gene-enriched pathways are selected using analytical formulas, and the number of differential genes in the enriched pathways is counted, with P<0.05 as the significant enrichment standard. Based on KEGG annotation results and classification, differentially expressed genes are categorized into biological pathways, and enrichment analysis is performed using the hyper function in software. Then, p-values are corrected for FDR, with functions usually considered significantly enriched at an FDR ≤ 0.01.

### Western blotting

An appropriate number of cryopreserved heart tissues and cells were collected, lysed in an ice bath, and homogenized. Extracted the supernatant and determined the total protein concentration of cells and tissues using the BCA method. After balancing, the protein was subjected to SDS-PAGE, transferred onto a membrane, sealed with 5% skim milk powder for 2 h [15], and incubated with primary antibodies (SHP2, p-PERK, MFN1, NLRP3, NOX2, NOX3, and GAPDH) on a horizontal shaker at 4° C overnight. After the membrane was washed, the protein was incubated again with secondary antibodies (1:5,000) on the horizontal shaker for 2 h at room temperature [18], the ECL imaging system was then used for color development and photography using a gel imaging system.

### Statistical analysis

Bioinformatics analysis was done using the DEseq2 and ggpubr packages of R software (v3.6.1). The Wald test was used for DEG analysis, and the rank sum test was used for cytokine comparisons between the two groups. Statistical analysis of other metrics was done using SPSS 23.0 software. Measurement data were expressed as mean ± standard deviation (χ±s). A one-way analysis of variance was adopted for comparison among groups, and an LSD test for pairwise comparison. p<0.05 was considered to be statistically significant.

## RESULTS

### Bioinformatics analysis

### Screening of DEGs

**Figure 1:**
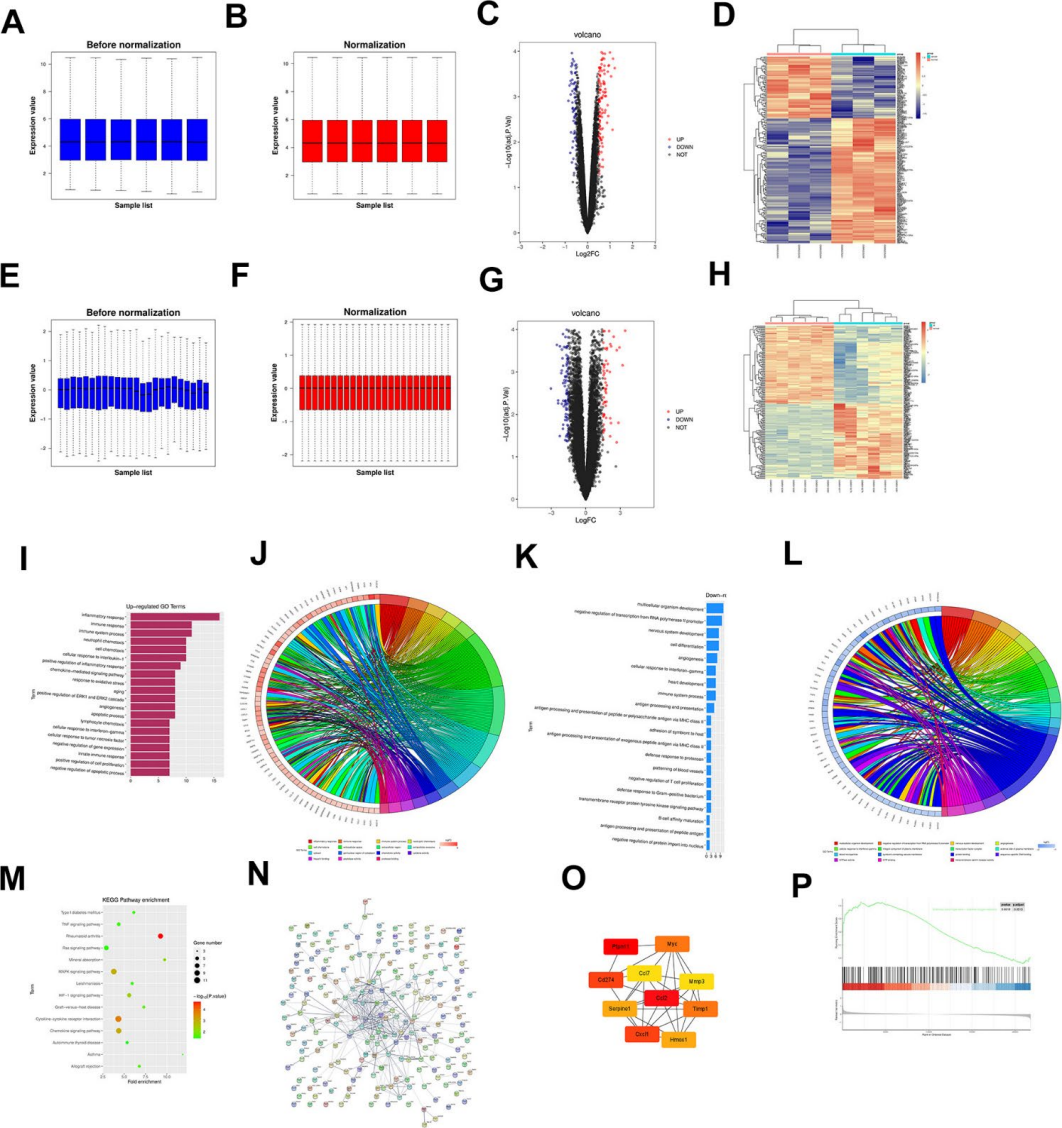
The dataset GSE171821 related to MIR was downloaded from the GEO database, and the data were subjected to quantile normalization (**A**, **B**). The volcano plot of visual grouping of DEGs in the dataset GSE171821 was constructed using the ggplot2 package of R software (**C**), and the cluster analysis heat map of DEGs was plotted using the pheatmap package of R software (**D**). Similarly, the dataset GSE108940 was subjected to quantile normalization (**E**, **F**). The volcano plot of visual grouping of DEGs in the dataset GSE108940 was constructed using the ggplot2 package of R software (**G**), and the cluster analysis heat map of DEGs was plotted using the pheatmap package of R software (**H**). The diagrams of up-regulated GO pathways (**I**, **J**) and down-regulated pathways (**K**, **L**) of DEGs were plotted using the R language. Then the DEGs were subjected to KEGG enrichment analysis, and the KEGG pathway diagram was plotted (**M**). The DEGs were imported into the STRING database to obtain the PPI network (**N**). Then the PPI network was imported into Cytoscape software, and the target genes with a score <10 were obtained using the plugin cytoHubba (**O**). GSEA was conducted on all genes using the clusterProfiler package of R software, and the GSEA pathway enrichment analysis diagram was also plotted (**P**).

Download the MIR-related dataset GSE171821 from the GEO database and quantile normalize the data (Figure 1A, 1B), followed by genetic screening (|logFC|<1 and p<0.05). From the results, it can be concluded that there are 104 DEGs in the mRNA of MIR, of which 102 are adjusted up and 51 are downward. A visually grouped volcano map of DEGs in the dataset GSE171821 was constructed using the ggplot2 package of R software (Figure 1C). A clustering heat map of DEGs was created using the Dynamic Plot package in R software. (Figure 1D). Also, the quantile normalization dataset GSE108940 (Figure 1E, 1F), and the DEGs were screened (|logFC|<1 and p<0.05). From the results, it can be observed that there are 104 DEGs in the mRNA of MIR, of which 34 are up-regulated and 70 are down-regulated. Using the ggplot2 package in R software, the volcano map of the visual grouping of DEGs in the dataset GSE108940 was constructed (Figure 1G). A clustering heat map of DEGs is plotted (Figure 1H).

### Bioinformatics analysis

The RobustRankAggreg software package of R software was used to cross-process GSE171821 and GSE108940 to obtain generic DEGs. The common DEGs were subjected to GO and KEGG enrichment analyses. The online database tool DAVID (https://david.ncifcrf.gov) was used to analyze DEGs at the bioprocess level, integrate GO terminology, and create a bioprocess network of DEGs. In addition, the up-regulation GO pathway (Figure 1I, 1J) and down-regulation pathways (Figure 1K, 1L) of DEGs were plotted using R. As can be seen from the diagrams, the up-regulated pathways included inflammatory response, immune response, and response to oxidative stress, and the down-regulated pathways included apoptotic, signal transduction, transcription and DNA-templated, which were all the enriched pathways of MIR. Then KEGG enrichment analysis was performed on DEGs and KEGG pathway maps were plotted (Figure 1M). It was found that the Ras signaling pathway, MAPK signaling pathway and cytokine-cytokine receptor interaction were enriched pathways.

### PPI network analysis of DEGs and screening of target genes

Import DEGs into the string database to obtain the PPI network (Figure 1N). The PPI network was then imported into Cytoscape software, and a target gene with a value of < 10 was obtained using the plug-in cell Hubba (Figure 1O).

### GSEA

GSEA detection is performed on all genes using the clustering analyzer software package of R software. The enrichment analysis of the GSEA pathway was plotted (Figure 1P). It was found that chemical carcinogenesis - reactive oxygen species was the enriched pathway.

### Allicin can reduce the myocardial infarction size in mice with ischemia-reperfusion

**Figure 2:**
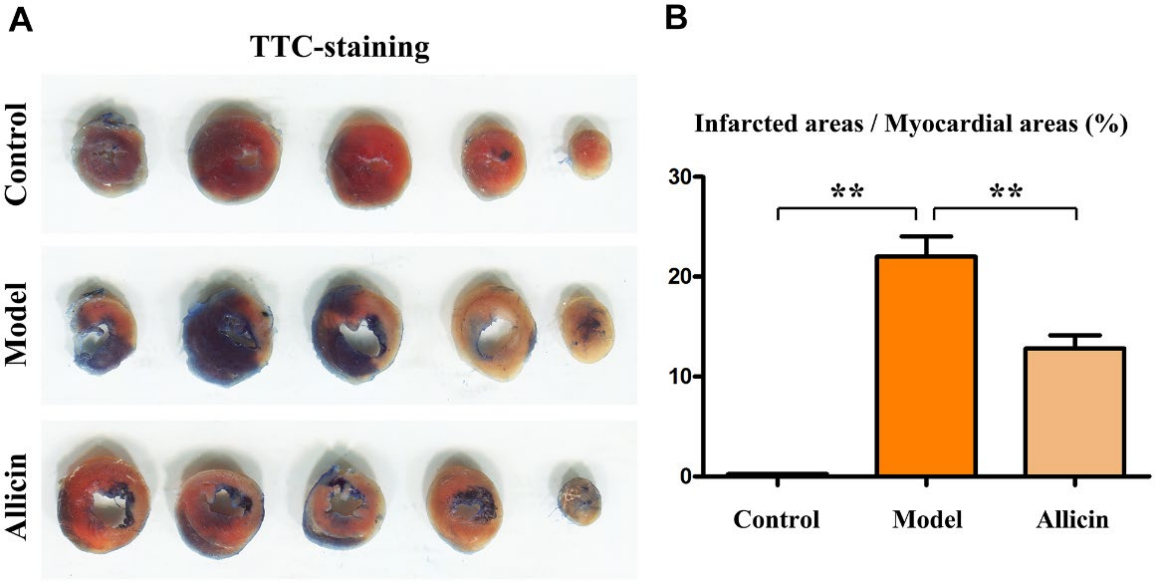
**Size of myocardial infarction in TTC-stained mice.** (**A**) TTC staining of the hearts in each group. (**B**) Statistical data on the ratio of TTC-positive area. One-way analysis, **p < 0.01, n = 6/group.

**Figure 3:**
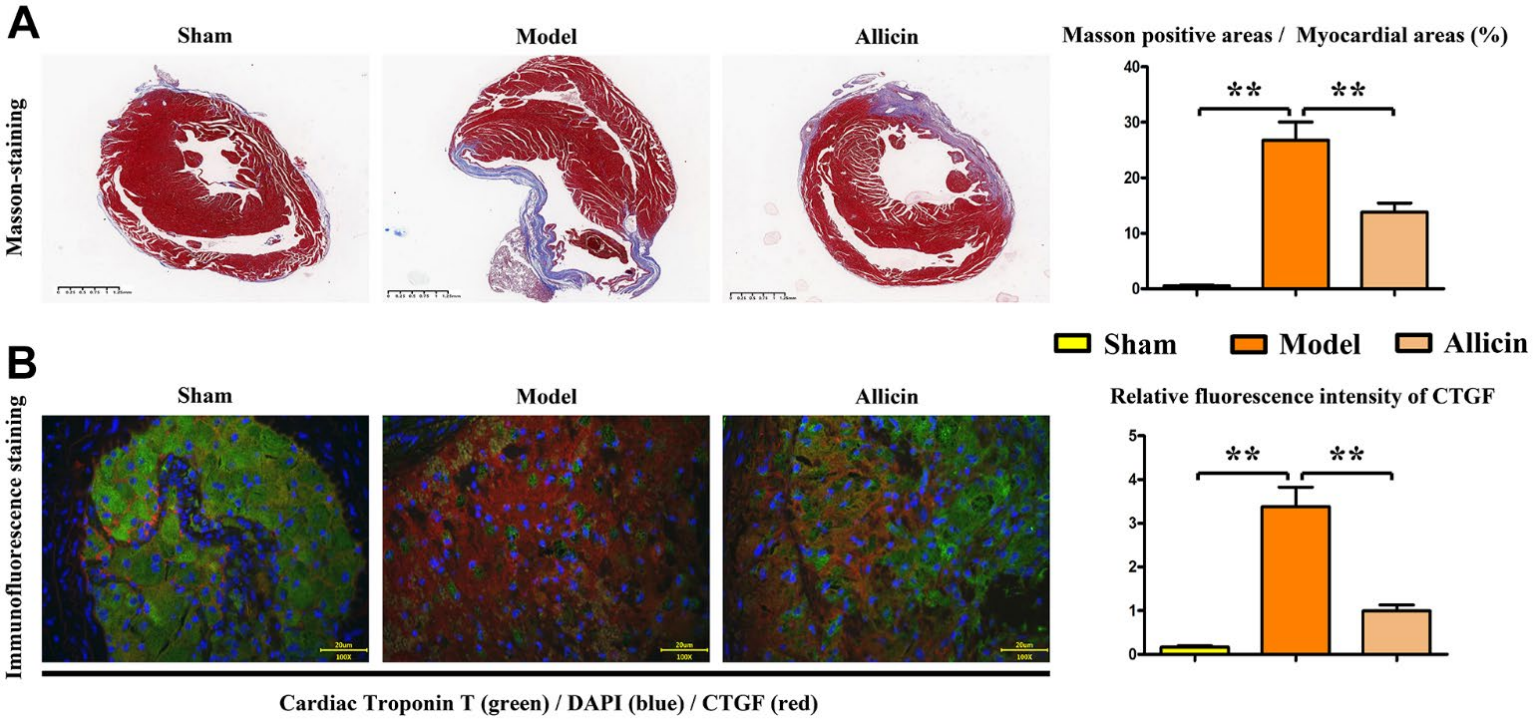
**Quantification of myocardial fibers and immunofluorescence staining of CTGF in Masson's-stained mice.** (**A**) Masson's staining of mouse hearts and the corresponding data. (**B**) Dual immunofluorescence staining of myocardial troponin T (T) and CTGF, and statistical analysis of CTGF intensity to evaluate the severity of cardiac fibrosis. One-way analysis, **p < 0.01, n = 6/group.

Firstly, we used TTC staining to assess whether the model was successfully constructed and if there was any noticeable morphological change with allicin treatment in the mouse hearts. The TTC staining showed that compared to the sham-operated group, the infarct area increased due to blood supply interruption in the I/R model group. Concurrent tissue edema and hemorrhage occurred, with an increase in the congested areas around the infarct perimeter (P<0.05). Compared to the I/R model group, the congested area and the infarct size were reduced in the group treated with allicin (P<0.05) (Figure 2). Subsequently, we used Masson’s staining to examine the condition of the myocardial fibers and observed that compared to the sham group, there was a significant increase in the proportion of diseased myocardial fibers relative to the infarct area in the I/R mice model. Edema and hemorrhage occurred simultaneously, with an increased congested area around the infarct (P<0.05). Compared to the I/R model group, the congested area and infarct size were reduced in the group treated with allicin (P<0.05) (Figure 3A). Then, we applied immunofluorescence techniques to detect troponin T and connective tissue growth factor (CTGF). Troponin T is widely used to diagnose and evaluate myocardial injuries, such as in the case of acute coronary syndrome (acute myocardial infarction), while CTGF stimulates fibroblasts to secrete collagen and other extracellular matrix molecules during fibrosis, promoting tissue deposition and scar formation. The immunofluorescence results showed that the levels of troponin T and CTGF were significantly increased in the I/R model group compared with the sham-operated group (P<0.05). In the group treated with allicin, there was a significant decrease in the expression of these markers compared to the I/R model group (P<0.05) (Figure 3B). These findings suggest that allicin can reduce the size of myocardial infarction in mice with ischemia-reperfusion at pathological, tissue, and molecular levels.

### Transcriptomic sequencing has revealed a significant upregulation of SHP2 *in situ* macrophages during the treatment of mouse myocardial ischemia-reperfusion with allicin

**Figure 4:**
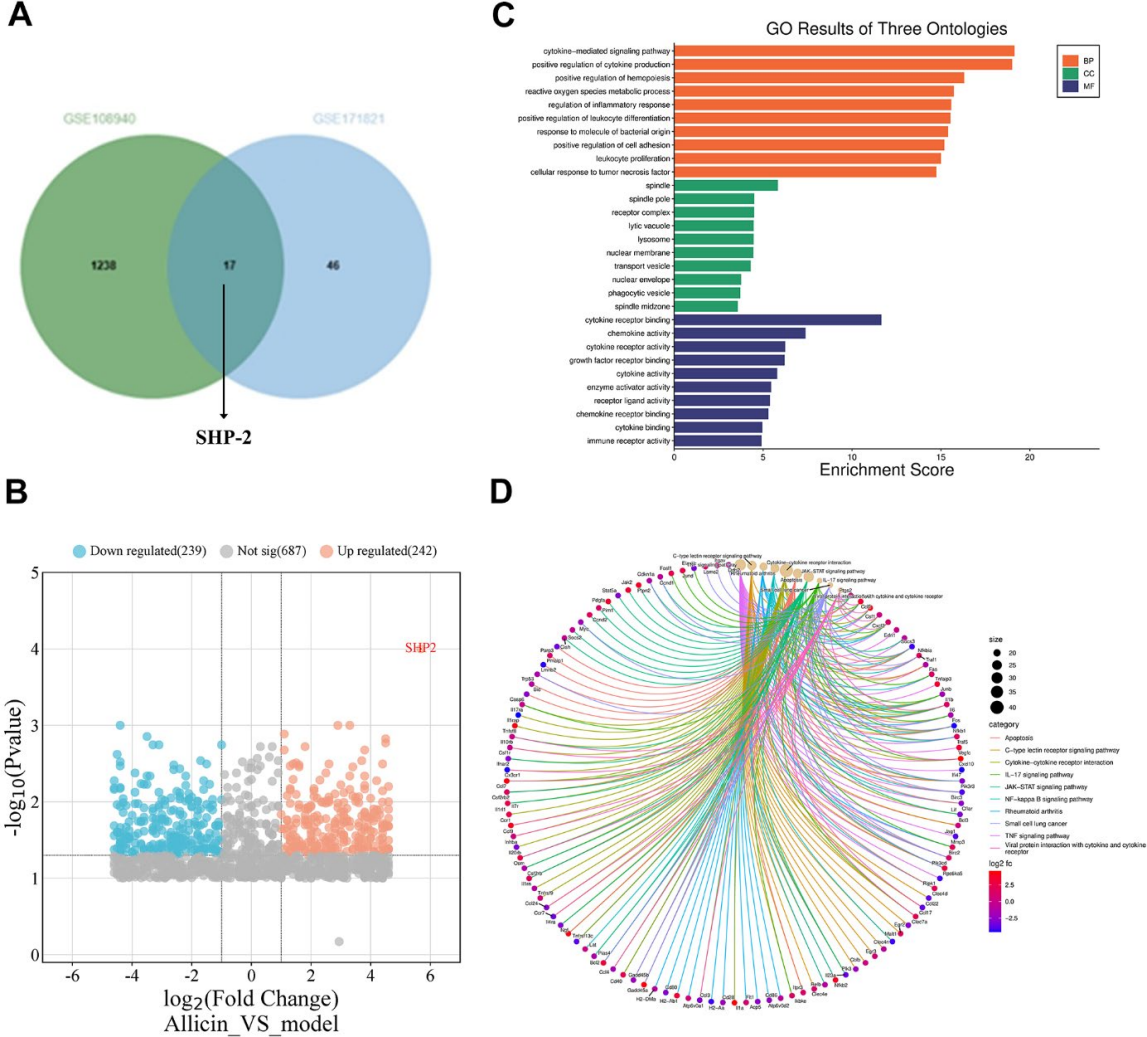
**Transcriptomic sequencing has revealed the gene expression profile of *in situ* macrophages during the treatment of myocardial ischemia-reperfusion in mice with allicin.** The gene SHP2 is significantly upregulated with the treatment of allicin, affecting the protection exerted by allicin in ischemia/reperfusion (I/R) conditions. (**A**) Intersection of datasets GSE171821 and GSE108940 revealed SHP2 as one of the critical genes within the intersection. (**B**) Volcano plots in heatmaps demonstrated the most significant difference in the expression of SHP2 mRNA in myocardial ischemia/reperfusion (MI/R) + allicin treatment. (**C**) GO analysis showed that kinase-mediated signaling pathways and oxidative stress-mediated signaling pathways had the most noticeable changes in allicin-treated mouse hearts under ischemia-reperfusion. (**D**) KEGG analysis indicated that virus-related macrophage signaling pathways were the most significantly altered in allicin-treated mice experiencing heart ischemia-reperfusion.

To elucidate the mechanism of the MI/R protection induced by allicin, we performed flow cytometry to sort CD68 positive cell populations from *in situ* macrophages of the heart in NC, MI/R, and MI/R+allicin groups, followed by transcriptomic sequencing analysis. The intersection of downregulated genes in GSE171821 and upregulated genes in GSE108940 identified SHP2 as one of the important genes within the intersection (Figure 4A). Volcano plots indicated that using criteria of |logFC| > 2 and Padj < 0.05, we obtained 242 upregulated differentially expressed genes and 239 downregulated genes (Figure 4B). GO analysis showed that kinase-mediated signaling pathways and oxidative stress-mediated signaling pathways had the most significant changes under allicin treatment during myocardial ischemia-reperfusion in mice (Figure 4C). KEGG analysis indicated that virus-related macrophage signaling pathways were the most significantly altered during myocardial ischemia-reperfusion under allicin treatment in mice (Figure 4D). This suggests that SHP2 and oxidative stress in macrophages play a very important role in the context of allicin treatment during myocardial ischemia-reperfusion in mice.

### Allicin can reduce the myocardial infarction size in mice with ischemia-reperfusion

Firstly, we utilized TTC staining of the mouse hearts to assess whether the model was successfully constructed and if there was a noticeable morphological change with allicin treatment. TTC staining revealed that, compared to the sham-operated group, there was an increased infarct area due to blood supply interruption in the I/R model group. Concurrent tissue edema and hemorrhage were observed, along with an increase in congestion areas around the infarct perimeter (P<0.05). Compared to the I/R model group, the congested area and infarct size were reduced in the I/R model group treated with allicin (P<0.05). (Figure 2) Next, we used Masson’s staining to assess the condition of myocardial fibers and observed that compared to the sham group, the proportion of diseased myocardial fibers relative to the infarct area was significantly increased in the I/R model group. Edema and hemorrhage occurred simultaneously, with an increased congestive area around the infarct (P<0.05). Compared to the I/R model group, the congested area and infarct size were reduced in the I/R model group treated with allicin (P<0.05) (Figure 3A). Subsequently, immunofluorescence techniques were used to detect myocardial troponin T and CTGF, the connective tissue growth factor. Troponin T is widely used for the diagnosis and evaluation of myocardial injuries such as acute coronary syndrome (acute myocardial infarction), while CTGF stimulates fibroblasts to secrete collagen and other extracellular matrix molecules during fibrosis, promoting tissue deposition and scar formation. Immunofluorescence results showed that troponin T and CTGF levels were significantly increased in the I/R model group compared with the sham-operated group (P<0.05). There was a significant decrease in the expression of these markers in the I/R model group treated with allicin compared with the I/R model group (P<0.05) (Figure 3B). These results indicate that allicin can reduce myocardial infarction size in mice with ischemia-reperfusion at pathological, tissue, and molecular levels.

### Allicin can enhance the expression of SHP2 in I/R mouse model

**Figure 5:**
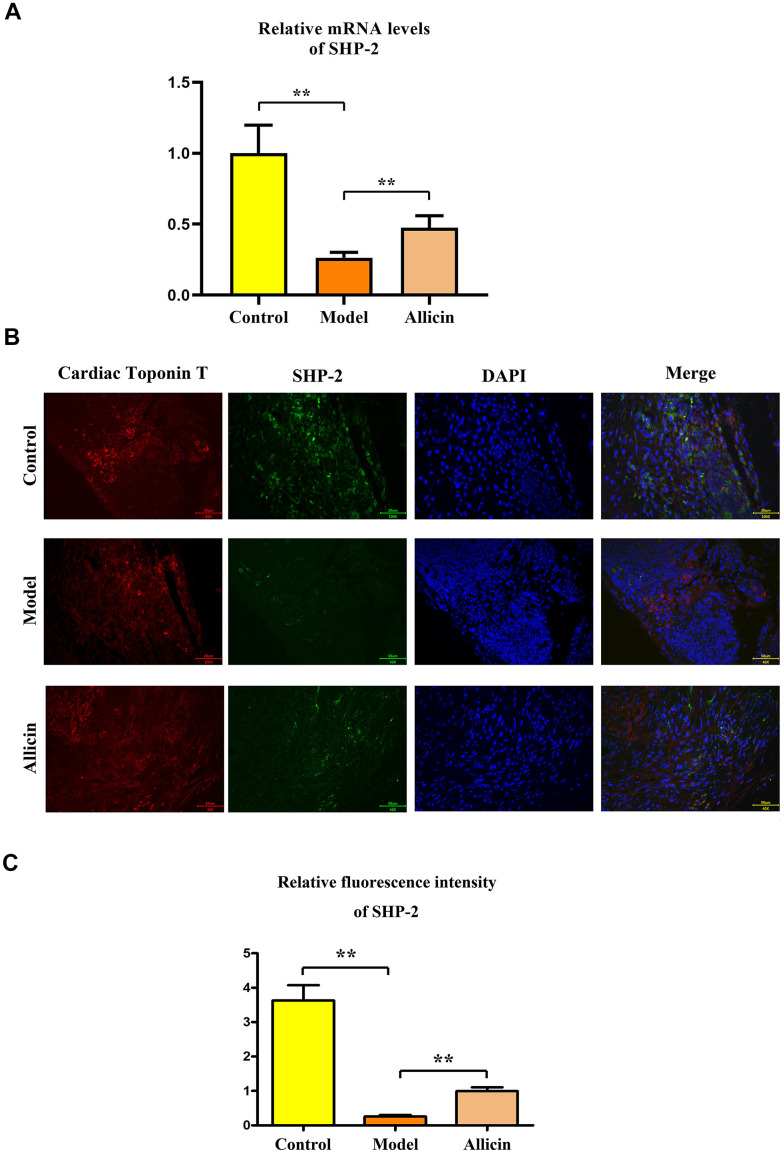
**Detection of SHP2 expression using RT-PCR and immunofluorescence.** (**A**) RT-PCR analysis of SHP2 mRNA expression, with corresponding statistical data. One-way analysis, **p < 0.01, n = 6/group. (**B**) Immunofluorescence staining for SHP2 expression and statistical analysis. One-way analysis, **p < 0.01, n = 6/group. (**C**) SHP2 fluorescence intensity statistical analysis. One-way analysis, **p < 0.01, n = 6/group.

**Figure 6:**
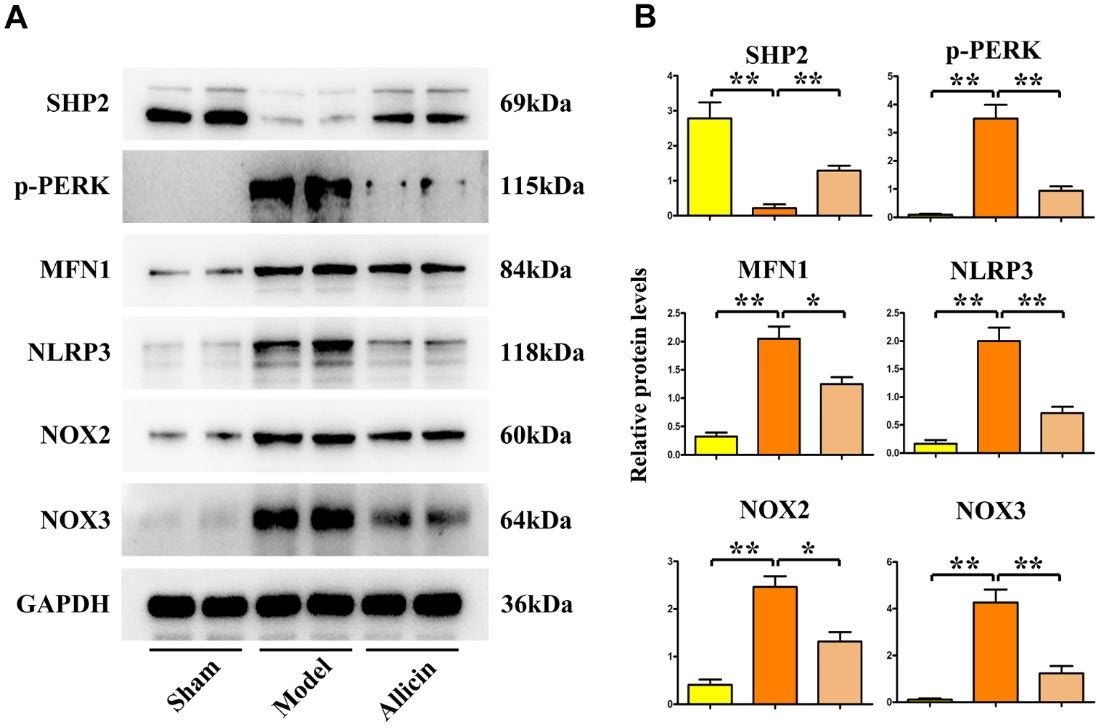
**Western blot analysis of allicin extract inhibiting mitochondrial oxidative stress.** (**A**, **B**) Allicin extract increased SHP2 protein expression and decreased the expression of p-PERK, MFN1, NLRP3, NOX2, and NOX3. **p < 0.01, One-way analysis, **p < 0.01, n = 6/group.

Through sequencing of the cardiac macrophages *in situ*, we selected SHP2 as our target gene for subsequent studies. RT-PCR and Western blot analyses showed a significant reduction in SHP2 mRNA (Figure 5A) and protein expression (Figure 6) in the I/R model group compared to the sham-operated group (P<0.05). Compared with the I/R model group, the expression levels of SHP2 mRNA and protein were significantly increased in the I/R model group treated with allicin (P<0.05) (Figure 5A). Immunofluorescence staining revealed that SHP2 immunofluorescence intensity was significantly weaker in the I/R model group compared to the sham-operated group (P<0.05). In contrast, SHP2 immunofluorescence intensity was significantly enhanced in the I/R model group treated with allicin, showing an opposite pattern to that of troponin T (P<0.05) (Figure 5B). This suggests that allicin can increase the expression of SHP2 in the I/R mouse model.

### Allicin ameliorated oxidative stress in heart tissues in I/R mice

GO and KEGG analyses from transcriptome sequencing indicated that an increased level of oxidative stress plays a key role. Western blot was used to detect the expression levels of proteins related to oxidative stress. Compared to the sham-operated group, the expression levels of p-PERK, MFN1, NLRP3, NOX2, and NOX3 proteins were significantly increased in the I/R model group (P<0.05). These levels were significantly reduced in the I/R model group treated with allicin (P<0.05) (Figure 6). Allicin decreased the level of oxidative stress in the I/R mouse model, and SHP2 was found to participate in oxidative stress.

### Allicin suppressed mitochondrial-oxidative stress production by increasing SHP2

**Figure 7:**
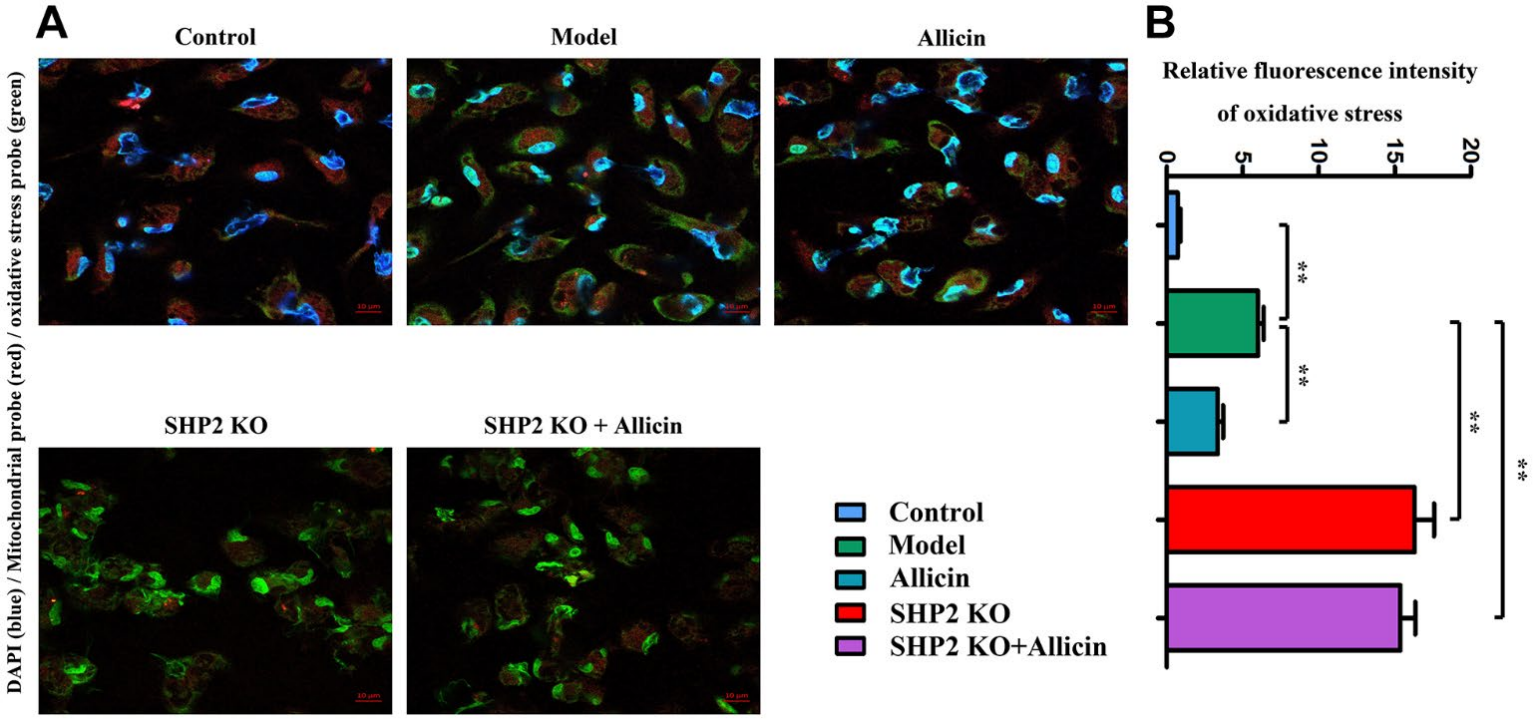
**Inhibition of macrophage oxidative stress by allicin extract.** (**A**) Allicin extract had an inhibitory effect on oxidative stress in SHP2-KO rats compared to the model group. Allicin extract had no significant effect on oxidative stress in SHP2-KO rats, indicating that mitochondria are the site of oxidative stress occurrence. (**B**) Statistical data. One-way analysis, **p < 0.01, n = 6/group.

We further verified whether allicin suppressed oxidative stress levels in I/R mice by increasing SHP2. I/R models were established using littermate controls and both SHP2-KO and wild-type (WT) mice: the sham-operated group (SHP-WT), I/R model group (SHP-WT), I/R model group treated with allicin (SHP-WT), I/R model group (SHP-KO), and I/R model group treated with allicin (SHP-KO). Oxidative stress and mitochondrial probes were used for immunofluorescence experiments. Results showed that ROS fluorescence intensity, indicative of increased oxidative stress, was higher in the I/R model group (SHP-WT) compared to the sham-operated group (SHP-WT) (P<0.05). ROS fluorescence intensity decreased in the I/R model group treated with allicin (SHP-WT) compared with the I/R model group (SHP-WT), indicating reduced oxidative stress levels (P<0.05). However, in the I/R model groups (SHP-KO) with or without allicin treatment, ROS intensity did not differ significantly compared with the I/R model group (SHP-KO) (P>0.05). This indicates that allicin can suppress oxidative stress in I/R mice through SHP2 (Figure 7).

### Allicin promoted expression of SHP2 and alleviated mouse I/R-induced oxidative stress through inhibiting activation of the p-PERK pathway

**Figure 8:**
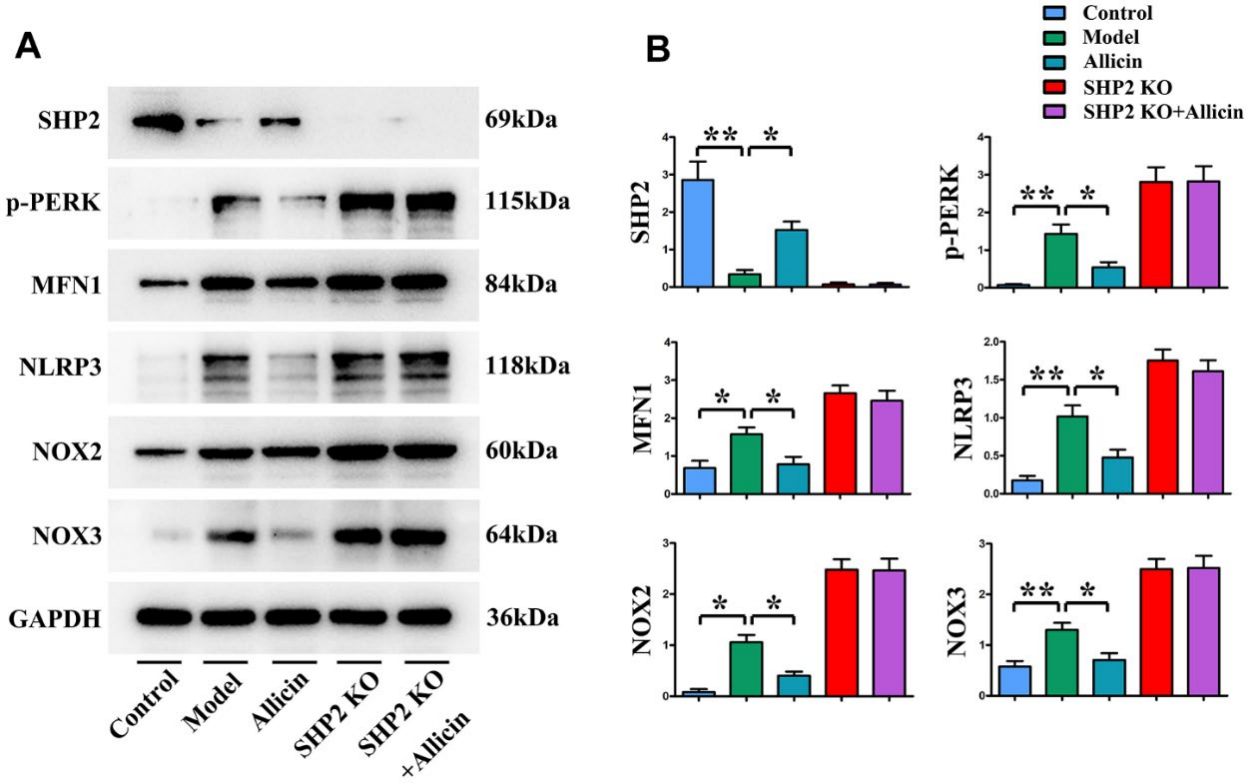
**Inhibition of oxidative stress by allicin extract through upregulation of SHP2 expression.** (**A**, **B**) Allicin extract inhibited the expression of p-PERK, MFN1, NOX2, NOX3, but increased SHP2 expression. Allicin extract did not affect oxidative stress in SHP2-KO rats. **p < 0.01, *p < 0.05, One-way analysis, **p < 0.01, n = 6/group.

Having demonstrated that allicin can suppress oxidative stress levels in I/R mice through SHP2, we sought to determine if this was affected by p-PERK, MFN1, NLRP3, NOX2, and NOX3. Pathway detection was carried out using heart tissues from the above models. Western blot experiments found that compared to the sham-operated group (SHP-WT), the expression of p-PERK, MFN1, NLRP3, NOX2, and NOX3 was significantly increased in the I/R model group (SHP-WT) (P<0.05). However, their expression levels were significantly reduced in the I/R model treated with allicin (SHP-WT) (P<0.05). The expression of these proteins was significantly enhanced in the I/R model group (SHP-KO) and the I/R model group treated with allicin (SHP-KO) compared to the I/R model group (SHP-WT) (P<0.05). No significant differences were found in the ROS levels when comparing the I/R model group treated with allicin (SHP-KO) with the I/R model group (SHP-KO) (P>0.05). This suggests that allicin promotes the reduction of oxidative stress levels in I/R mice through the p-PERK pathway by enhancing the expression of SHP2 (Figure 8).

## DISCUSSION

Reducing reperfusion injury is key to improving outcomes in patients with acute myocardial ischemia, which is commonly achieved by pre-treatment medication in the clinic [19]. As a kind of diallyl trisulfide with multiple biological activities, allicin, through improving the hemodynamic indexes (increasing the maximum rate of increase in ventricular pressure and the maximum rate of decrease in left ventricular pressure, and improving the coronary blood flow) and the activity of superoxide dismutase, can suppress oxidative stress injury and inflammatory response, and reduce sodium current, thereby exerting a certain protective effect against MIRI. At present, allicin has been primarily used for the clinical treatment of deep fungal and bacterial infections. In recent years, a good anti-fibrotic effect of allicin has been verified in pharmacological studies. Numerous studies have found that allicin has important functions such as antioxidant, antitumor, and bacteriostatic effects. At present, steam distillation, solvent extraction, and supercritical extraction are the main extraction methods for allicin. Although a lot of research has been done on allicin, large-scale production and widespread application of allicin products remain to be achieved [18, 20, 21]. The results of this study showed that allicin could significantly reduce the area of myocardial infarction and myocardial fibrosis after myocardial injury in mice. Then, the role of allicin in MIRI in mice was further investigated. However, the pharmacological effects of allicin may not be particularly comprehensive in this study, so its pharmacological mechanism remains to be further explored.

MIRI involves many factors, and its occurrence, development, and prevention are associated with oxidative stress [22]. There is a study that MAM is an important player in energy generation, cell contraction and movement, and intracellular/extracellular signal transmission, and it can be implicated in such biological processes as the production and accumulation of mitochondrial ROS, release of downstream inflammatory factors, regulation of autophagy, endoplasmic reticulum stress and programmed cell death [17]. In mammals, the fusion of the outer mitochondrial membrane is dependent on MFN, including MFN1 and MFN2, which can stabilize the interaction between adjacent mitochondria and the interrelation between mitochondria and endoplasmic reticulum, and regulate the mitochondrial morphology [23]. In this study, it was found that MIRI may affect the regulatory relation between mitochondria and endoplasmic reticulum. Allicin could down-regulate the protein expression of MFN in heart tissues in MIRI mice, and the protein expressions of NLRP3, NOX2, and NOX3 also declined. Research suggests that NADPH, also known as the NOX family, is a protein that transfers electrons on biological membranes, and its main biological function is to generate ROS [24, 25]. NLRP3 inflammasome is a multi-protein complex that can be activated by K+ efflux, Ca^2+^ overload, ROS release, endoplasmic reticulum stress, lysosomal leakage, and mitochondrial dysfunction, the first two of which are important mechanisms for its activation [26]. After MIRI occurs, there will be massive Ca^2+^ influx and ROS accumulation in rats, and such changes will trigger the activation of NLRP3 inflammasome [27] and mediate the occurrence of inflammatory response [26, 28], leading to a pathological response. The close association between the activation of NLRP3 and the generation of ROS is also confirmed in some studies. The results showed that allicin inhibited the activation of NLRP3, thereby improving the oxidative stress response of MIRI.

According to bioinformatics analysis, SHP2 had a low expression in heart tissues in I/R mice, suggesting the important role of SHP2 in the occurrence and development of MIRI in mice. It was found that in I/R animal models and macrophages, the expression of SHP2 in the model group was lower than that in the control group, consistent with the results of bioinformatics analysis. Moreover, allicin raised the protein expression of SHP2 in the model group in animal and cell experiments. As proved by studies, the ablation or inhibition of SHP2 in macrophages can enhance the activation of NLRP3 [29, 30], indicating that SHP2 is a negative regulator of NLRP3 inflammasome, consistent with the findings in this study. In addition, the expression of SHP2 was significantly increased in cSHP2-KO mouse-derived macrophages compared with that in WT mouse-derived macrophages. There was no significant difference in the expression of proteins in the SHP2 KO + allicin group and those in the SHP2 KO group. The results showed that the inhibition of SHP2 cut off the effects of allicin on p-PERK and NLRP3, indicating that allicin inhibited the occurrence of oxidative stress after activating p-PERK in I/R mice by promoting the expression of SHP2 and inhibiting the activation of p-PERK. Moreover, the oxidative stress-related factors P22, P47, and gp91 were detected in each group by qPCR. It was found that the expressions of P22, P47, and gp91 in myocardial I/R mouse models significantly declined after the use of allicin, which, combined with *in vitro* cell experiments, suggested that allicin can mediate the SHP2 axis to reduce the level of oxidative stress in MIR. It is speculated that allicin can regulate SHP2 by mediating p-PERK, thereby inhibiting MIRI-induced oxidative stress. Other studies have found that allicin can regulate angiogenesis in MIR through other pathways, such as miR-19a-3p/PI3K/AKT pathway, indicating that allicin can play different roles through different signaling pathways. These findings protect MIRI [16].

In summary, allicin can inhibit the expression of p-PERK by increasing the expression of SHP2, thereby inhibiting the level of oxidative stress in myocardial ischemia-reperfusion mice.
